# Frequency of prescriptions of off-label drugs and drugs not approved for pediatric use in primary health care in a southern municipality of Brazil

**DOI:** 10.1016/j.rppede.2015.06.023

**Published:** 2016

**Authors:** Marcele Giacomin Gonçalves, Isabela Heineck

**Affiliations:** Faculdade de Farmácia, Universidade Federal do Rio Grande do Sul (UFRGS), Porto Alegre, RS, Brazil

**Keywords:** Pediatrics, Medication use, Primary health care

## Abstract

**Objective:**

To determine the frequency of prescriptions of off-label drugs and drugs not approved for pediatric use in primary health care in medium-sized municipality of Rio Grande do Sul, Brazil.

**Methods:**

Cross-sectional study with retrospective data collection, which analyzed prescriptions issued to 326 patients from August to December/2012 in two basic health units in the city of Viamão, state of Rio Grande do Sul. It included all prescriptions of patients whose medical records or service records were available and complete in relation to the date of presence, weight and date of birth. Off-label prescriptions were those which, in relation to the drug leaflet, showed dose different the recommended range, frequency of prescription and/or different form of administration and younger age than the indicated range. Descriptive statistics with absolute frequencies, means and standard deviations were used.

**Results:**

During the study period, a total of 731 drug prescriptions were issued and the frequency of off-label medications prescribed was 31.7%, especially antihistamines and antiasthmatics (32.3% and 31.5%, respectively). The main type of off-label prescription was dose (38.8%), followed by age range (31.5%) and frequency of administration (29.3%). Regarding the dose off-label prescription, overdose was more frequent (93.3%) than the underdose (6.7%). Prescriptions of unapproved drugs were not identified.

**Conclusions:**

The study showed that off label prescription is common in both assessed units. The observed percentage of off label prescription was higher than that reported by European studies carried out in primary care. On the other hand, the prescription of drugs not approved for children was not observed.

## Introduction

Considering the lack of drugs for use in children, especially those under 2 years of age, the off-label prescription drugs have become a routine practice both in hospitals and ambulatory and brings doubts to prescribers and providers regarding the benefit to the pediatric patient.^1^^,^ 2

The term “off-label” refers to drugs prescribed in different manner than that directed in the instructions or official compendia in relation to dose, indication, age group, dosing interval, or form of administration.^3^ Off-label prescription is not illegal; it is not necessarily incorrect and is present in several pediatric protocols. The quality of drug therapy is not necessarily related to the licensing status of the drug. However, there are several clinical, ethical, and safety factors that should be considered and there are no guidelines to assist off-label prescription. The decision on this type of prescription should be assessed according to clinical indication, treatment options, and risk-benefit analysis. Moreover, it must obtain the patient's or guardian consent, taking care to avoid exposing children to unnecessary risks.^4^

Regarding the concept of unlicensed medicine, some authors consider that it refers to drugs that are unregistered in the surveillance agency, or are extemporaneous preparations, or drugs containing non-pharmacological chemical ingredients used with therapeutic purpose.^5^^–^ 8 Ferreira et al. extend the concept of unlicensed to registered drugs that are contraindicated for children.^9^

There are studies that characterize the extent of off-label prescribing in pediatric hospitals in Brazil,^9^^–^ 11 but little is known of outpatient prescription in primary care. This study aims to fill this gap, determine the frequency of prescription of off-label and unlicensed drugs for pediatrics, to support the development of actions to promote rational use of drugs.

## Method

Cross-sectional study with retrospective data collection, approved by the Research Ethics Committee of the Universidade Federal do Rio Grande do Sul (No. 214 535) and authorized by the Municipal Secretary of Health of Viamão. Data collection was performed in two basic health units: Family Health Strategy (FHS) Itapuã and Reference Unit (RU) Lomba Sabão, Viamão. Viamão is a city of the metropolitan area located 20.6km from the capital Porto Alegre, with an area of 1494.26km^2^ and 239,384 inhabitants, according to the Demographic Census of the Brazilian Institute of Geography and Statistics (IBGE) 2010.^12^

Sample size calculation was performed considering 3759 pediatric consultations in health facilities involved during a period of five months (August–December 2012). A 20% expected frequency of off-label prescription drugs was used.^4^^–^ 8 ^,^ 13 ^–^ 16 To a 95% confidence interval, a 20%+5% range was considered. Thus, it was estimated that at least 231 prescriptions should be evaluated.

Copies of prescriptions retained in the unit dispensary after dispensing were evaluated, with medication of patients under the age of 12 attended by pediatricians of the respective health units, from August to December 2012. We excluded prescriptions for patients not linked to the unit. Unit user prescriptions whose medical records were not found or were incomplete regarding the variables of interest were not considered.

Prescription data were recorded on a specific table and supplemented with information from medical records or patient's chart. The variables of interest related to the patient (age, sex, and weight) and prescriptions (total items, prescription drugs, presentations, dosage form, route of administration, frequency of administration, and dose) were recorded.

For data analysis patients were divided into four groups according to age: infants (0–2 years), preschoolers (>2–7 years), schoolers (>7–10 years), and adolescents (>10–12 years). The prescribed drugs were classified according to ANVISA Electronic Labeling (Bulário Eletrônico da Agência Nacional de Vigilância Sanitária)^17^ and, in the absence of information on this site, instructions provided by the manufacturer into three classifications: according to specification (age, dose, frequency of administration and form of administration, as specified in the package insert); off-label (drugs prescribed for different ages, at higher or lower doses, with different dosing frequency and administration manner other than the indicated)^18^; and not licensed for children (drugs for which there was no information or were contraindicated for children,^9^ registration and labeling only considered the adult use).

The Anatomical Therapeutic Chemical Classification (ATC)^19^ was used to enable the analysis of drugs by therapeutic classes. Data were organized in Microsoft Office Excel 2007 spreadsheet and analyzed using the SPSS 18.0 software. Descriptive statistics with absolute frequencies (mean and standard deviation) was used.

## Results

Prescriptions for 705 pediatric patients were retained in the study period in selected units, generally more than one prescription per patient. Only 326 of these prescriptions were included in the study, as 379 records were not found or were incomplete. Regarding care units, 203 (62.3%) patients were from the Family Health Strategy (FHS) Itapuã and the other 123 (37.7%) from the Reference Unit (RU) Lomba Sabão. Of the 326 children, 56.4% were male. There was a higher prevalence of infants, 142 (43.3%); followed by preschoolers, 103 (31.6%). Schoolers 55 (16.9%) and adolescents 27 (8.3%) were minority and amounted to 25.2% of the patients. Among infants (0–2 years), 89 (63%) were up to 12 months old and 53 (37%) from 13 months to 2 years old. The number of drugs ranged from one to eight, with an average of 2.2±1.4 per patient. According to the analysis of prescriptions, 95.4% of all prescribed drugs belonged to the municipal list of essential medicines (RESUME).

In total, 39 different active principles (30 isolates and 9 combinations) were prescribed in different presentations. The most frequently prescribed drugs were: paracetamol 88 (11.8%); nasal saline 81 (11.1%); loratadine 80 (10.3%); amoxicillin 66 (8.3%); prednisolone 60 (8.2%), and oral spray salbuterol 53 (7.3%). Table 1 describes the frequency of prescription by therapeutic class.

**Table 1:** Therapeutic class of prescribed drugs and prescription frequency.

ATC group	Class	Drugs (number of prescriptions)	Prescriptions, n (%)
N02	Analgesics, antipyretics	Paracetamol (88), dipirone (7), ibuprofen (26) a	121(16.6%)
R03	Drugs used in obstructive respiratory diseases	Beclomethasone (40), disodium cromoglycate (1), fenoterol (18), salbutamol (53), budesonide (1)	113 (15.5%)
R01	Nasal preparations	Budesonide (4), sodium chloride (81), beclomethasone (24)	10 (14.9%)
R06	Systemic antihistamines	Loratadine (80), dexchlorpheniramine (8), brompheniramine (1)	89 (12.2%)
J01	Systemic antimicrobials	Amoxicillin (66), cephalexin (6), sulfamethoxazole+trimethoprim (2), metronidazole (1)	75 (10.3%)
H02	Systemic corticosteroids	Prednisolone (60), prednisone (12)	72 (9.9%)
P02	Antiparasitics, anthelmintics	Albendazole (19), ivermectin (1), mebendazole (12)	32 (4.4%)
A03	Drugs used in gastrointestinal disorders	dimethicone (18), metoclopramide (7), bromopride (4)	29 (4.0%)
B03	Antianemics	Ferrous sulphate (21)	21 (2.9%)
D07	Topical corticosteroids	Dexamethasone (17), desonide (1)	18 (2.5%)
D01	Topical antifungal	miconazole (11)	11 (1.5%)
A011	Vitamins	Vitamin A+D (10)	10 (1.4%)
D06	Topical antimicrobials	Neomycin+bacitracin (8)	8 (1.1%)
S02	Otologic drugs	Borate 8-hydroxyquinoline trolamine (5), neomycin + polymyxin+hydrocortisone (1)	6 (0.8%)
A01	Stomatological preparations	Nystatin (5)	5 (0.7%)
P03	Ectoparasiticides/scabicides	Permethrin (4)	4 (0.6%)
A07	Antidiarrheals, antimicrobial agents	Oral rehydration salts (3), *Saccharomyces boulardii* (1)	4 (0.6%)
A06	Constipation drugs	Lactulose (2)	2 (0.3%)
M01	Antiinflammatory	Nimesulide (1)	1 (0.1%)
D02	Emollients and protectors	Zinc oxide (slurry water) (1)	1 (0.1%)

a Ibuprofen prescribed as an analgesic and antipyretic.

ATC, anatomical therapeutic chemical classification.

We found 731 prescribed drugs. There was no prescription of unlicensed drugs for children. All prescribed drugs were for adult and pediatric use. Of the total, 232 (31.7%) prescriptions were off-label and 13 (0.02%) had no specification of the prescribed dose, only the name of the active ingredient. Drugs not specifying the dose were: vitamin A+D and permethrin (for 9 [1.2%] and 4 [0.6%] children, respectively). The classification of prescribed drugs is summarized in Table 2.

**Table 2:** Classification of prescribed drugs regarding its package insert specification.

Classification	Frequency, n (%)
According to specification	486 (66.5%)
Unlicensed	0
Off-label prescribing	232 (31.7%)
Unable to classify a	13 (1.8%)
Total drugs prescribed	731 (100%)

a Unable to classify because there was no pharmaceutical form on prescription.

Among the off-label prescriptions, the following types and frequencies were seen: off-label dose, 90 (38.8%); followed by age, 73 (31.5%); and frequency of administration, 68 (29.3%). Regarding off-label dose, overdosing was more prevalent than underdosing: 84 (93.3%) vs. 6 (6.7%) prescriptions, respectively.

The off-label prescribed drugs are presented in Table 3 by therapeutic classes. Among them, we highlight loratadine, oral spray salbutamol, fenoterol, and dimethicone.

**Table 3:** Type of off-label use by therapeutic class.

ATC group	Class	Dose (n=90)	Age (n=73)	Dosing interval (n=68)	Form of administration (n=1)
R06	Systemic antihistamines	17 (7.3%)	22 (9.5%)	36 (15.5%)	
R03	Drugs used in obstructive respiratory diseases	28 (12.1%)	30 (12.9%)	15 (6.5%)	
J01	Systemic antimicrobials	25 (10.8%)			
A03	Drugs used in gastrointestinal disorders	2 (0.9%)	7 (3.0%)	15 (6.5%)	
R01	Nasal preparations	1 (0.4%)	8 (3.5%)	1 (0.4%)	
N02	Analgesics, antipyretics	5 (2.2%)	3 (1.3%)		
B03	Antianemics	4 (1.7%)			1 (0.4%)
H02	Systemic corticosteroids	3 (1.3%)			
P02	Antiparasitics, anthelmintics	2 (0.9%)			
M01	Anti-inflammatory	1 (0.4%)	2 (0.9%)	1 (0.4%)	
S02	Otologic drugs	2 (0.9%)	1 (0.4%)		

Percentage of total off-label prescribing (232); ATC, anatomical therapeutic chemical classification.

Loratadine was the third most prescribed drug and had a frequency of off-label prescription of 85.3%, dosing frequency of 53.1%, younger age than the recommended of 25%, and overdosing of 21.9%. Regarding prescriptions for off-label frequency of administration, 19 (55.9%) were also off-label dose; that is, two types of discordant recommended use.

Salbutamol, whose frequency of prescription was 7.3% (53) was prescribed off-label in 100% of prescriptions: indicated for an age group younger than the recommended in 27 (50.9%) and for use in doses higher than recommended in the package insert in 26 (49.1%) cases.

Fenoterol had 18 prescriptions. Only one of them was in accordance with the package insert, the other 17 (94.4%) were classified as off-label. Of these, 15 (88.2%) were off-label for frequency of administration above the recommended and two (11.8%) due to the higher dose than recommended. The two prescriptions with dose higher than recommended also had frequency of administration above the recommended.

Dimethicone, a medication out of the municipal list of essential medicines, was prescribed in 17 occasions, 16 of them (94.1%) off-label: 14 for frequency of administration above the recommended and twice for overdosing. In both cases of overdosing there was also the incorrect prescribing of frequency of administration.

In the analysis of off-label prescribed drugs by age group, it is noteworthily the prescription of loratadine and dexchlorpheniramine for infants—an age group for which these drugs are not recommended. In the group of preschoolers, it is worth noting the prescription of salbutamol and amoxicillin at doses higher than recommended. In the age groups corresponding to schoolers and adolescents, the overdose of salbutamol was what stood out.

## Discussion

There was no prescription of unlicensed drugs. Other authors found percentages, which varied from 0.3 to 16.8%.^5^^,^ 14 ^,^ 18 The absence of unlicensed drugs is relevant and may be related to the high adherence (95.4%) of Viamão pediatricians to the list of essential medicines in the municipality (RESUME). A lower percentage of adherence to the RESUME (76.4%) was seen in a study performed in eight cities of three Brazilian states.^20^ The use of lists of essential medicines is a measure recommended by the World Health Organization to promote the rational use of drugs.^21^ The availability of a smaller therapeutic arsenal and that takes into account the health needs of the majority of the population can reduce the chance of using unlicensed products.

On the other hand, the prescription of off-label drugs in primary care in Viamão was high, with a frequency of 31.7% above the range found in other European population-based studies: Scotland (24.6%), England (16%), Netherlands (20.3%), Estonia (31%), Italy (17%), and France (29%).^6^^,^ 8 ^,^ 13 ^,^ 15 ^,^ 22 ^,^ 23 The main types of off-label prescription in this study were dose (38.8%), age (31.5%), and frequency of administration (29.3%). Similarly, the above-mentioned European studies indicated dose and age as the main types of off-label use, in that order. The frequency of drug administration was not among the off-label types, which probably is due to the fact that other authors evaluated the dose and frequency of administration together and classified the cases out of specification as off-label dose.

Overdosing was more frequent, unlike reported by other authors who found a greater number of underdosing, particularly for the class of antimicrobial drugs for systemic use.^13^^,^ 14 ^,^ 24 There was a prevalence of 10.3% of antimicrobial prescriptions, which represented 10.8% of total off-label prescriptions, mostly by overdosing. This result shows a different trend from that of other studies associating underdosing with physicians difficulty to adjust the dose to the child's age, that is, to know the age and certain situations in which doses should be increased.^24^ Amoxicillin, the most prescribed antimicrobial in this study, has a dose indication on label less than that of other sources, such as the Formulário Terapêutico Nacional.^25^ The data suggest a downgrade of the package insert published in the ANVISA Electronic Labeling and reinforce the need to constantly update this source of information on the part of manufacturers and the product license review by the agency. Another important measure would be to establish a municipal Pharmacy and Therapeutics Committee aiming at the development of protocols for the use of the medicines in the list according to recent studies, to support the prescriber and promote the rational use of medicines. It is worth remembering that the quality of drug therapy is not necessarily related to the drug's regulatory status.

The most commonly prescribed off-label drugs were the anti-asthmatics and antihistamines for systemic use (Table 3). These classes of drugs are among the most commonly prescribed in pediatric primary care.^6^^,^ 16 In this study, loratadine was prescribed off-label in 85.3% of cases regarding frequency of administration (53.1%), lower than recommended age group (25%), and overdosing (21.9%). Considering that the drug's plasma half-life is 17–24h,^25^ there would be no need or indication for multiple daily doses, as observed in this study. The use of antihistamines in a way not appropriate for the age has been reported in recent literature review that showed a variation of 6.5–43%.^26^ Da Costa et al. reported Loratadine as one of the drugs hard to deal with in pediatrics due to the restriction for age under 2 years.^1^ The usual of Loratadine is 5mg for children 2–6 years old and under 30kg and 10mg once-daily for children over 6 years old and adults.^25^^,^ 26 The study showed overdosing in prescriptions of loratadine. Only one study advocated the use of a higher dose than that required in the package insert for treating allergic asthma.^27^ Considering that such information is not confirmed in the 2012 Guidelines of the Brazilian Society of Pneumology and Phthisiology for the Management of Asthma (Sociedade Brasileira de Pneumologia e Tisiologia para o Manejo da Asma), it is evident the need for continuing education of professionals and dose standardization based on clinical trials and, if none, observational studies.^28^

There was a prevalence of off-label prescribing of 31.5% for respiratory disease management (Table 3), salbutamol spray and fenoterol nebulizer solution standed out among the medications. Treatment of asthma in children is challenging and often an overlap between recurrent wheezing and asthma phenotypes occurs, making diagnostic and therapeutic decisions controversial.^28^ Recommendations of the Brazilian Society of Pneumology and Phthisiology (SBPT) differ from the package insert recommendations for salbutamol spray. The guidelines indicate the use in children over 5 years old and with higher doses than those indicated by the manufacturer, even with possible limitations to the correct use of the device in this age group. The drug package insert highlights the difficulty of the device correct use in children under 7 years, with no restrictions for use above this age group. Considering the large number of hospitalizations and the risks of non-treatment of an asthma attack (suffocation is the leading cause of death in almost all cases),^28^ adapting the adult dose for children is a recurring practice based on clinician's knowledge, but with little documentation by Brazilian physicians.^25^ Although the clinical benefit of these drugs in acute asthma management is well documented, there is great variability in the doses used, mostly based on expert opinion, clinical consensus, or studies with a limited number of patients. Little evidence precisely support the doses to be used.^28^

The use of salbutamol, a beta-adrenergic agonist, in high doses is associated with tremors, agitation, hypokalemia, and cardiac arrhythmias. There is evidence that treatment with salbutamol shifts the cardiovascular autonomic regulation to a new level, characterized by a greater sympathetic response and mild β2-receptor tolerance. Based on this evidence, salbutamol abuse may be a substrate for atrial fibrillation.^29^

The most important determinant of the daily dosage is clinical judgment of the patient's response to treatment. The doctor should monitor the patient's response and adjust the dose according to the level of asthma control. Low, medium or high doses settings are based on manufacturers’ pharmacokinetic and pharmacodynamic studies, which are rarely based on dose-response curves. They vary according to the device used and must be assessed individually.^28^

It is noteworthy the fact that some prescriptions had no dosage, only the name of the drug. For the patient, prescription is an important support in the treatment and to be effective it should have the basic items, including the dosage.^30^

Most medicines used by children are prescribed in primary care, and pediatricians or general practitioners use a relatively small number of drugs to solve the most common problems. The small cast of medicines facilitates the development of municipal clinical protocol, built in a multidisciplinary way in the Pharmacy and Therapeutics Committee. This contrasts with secondary care in which the number of children is lower compared to the general population, but a much larger number of drugs is prescribed to treat rare and more serious conditions.^13^

Extrapolation of this study's results should be done with caution, as data refer to two health units, a five-month period, and half of the prescriptions could not be assessed due to lack of information. Prescribing habits vary according to the formation of pediatricians, protocols, or even service routines. Studies involving a larger number of health units are needed to establish more accurately the extent of off-label prescribing in municipal or regional level. Furthermore, the limitations inherent in retrospective studies should be considered. Information was collected from prescriptions retained in pharmacies and manual records. The incomplete record of the name of patients and the different criteria of cataloging records determined the exclusion of some cases. The retrospective data collection also prevented the analysis of off-label usage by indication, as this data was rarely found in medical records or patients’ charts. We should also consider that only the prescriptions dispensed are retained in the pharmacies of these units. Patients may have received other prescription of drugs subject to special control, not present in REMUME, or of special or specialized component of the pharmaceutical care. Despite these limitations, this study brings contributions, as the Brazilian data on the use of unlicensed and off-label medicines in children are so far restricted to hospitals.

The difficulties related to research with children foster off-label prescribing. Although this practice is not illegal, it creates uncertainty regarding the possible adverse effects in a population with specific characteristics such as the pediatric population. The present study showed that this practice is common in primary health care in a city of Rio Grande do Sul, similar to studies in European cities. We hope that the results may contribute to the planning of actions to support prescribers and provide greater security in the use of medicines for pediatric patients.
