# Supplementary material for: Frequency of prescriptions of off-label drugs and drugs not approved for pediatric use in primary health care in a southern municipality of Brazil
Source: Rev Paul Pediatr. 2016 Jan-Mar;34(1):11–7. doi: 10.1016/j.rppede.2015.06.023 (PMC4795716; doi:10.1016/j.rppede.2015.06.023)
Supplement: Translation

# Translation: Frequência de prescrições de medicamentos *off label* e não licenciados para pediatria na atenção primária à saúde em município do sul do Brasil

**Authors:** Marcele Giacomin Gonçalves, Isabela Heineck

**Affiliations:** Faculdade de Farmácia, Universidade Federal do Rio Grande do Sul (UFRGS), Porto Alegre, RS, Brasil

**Keywords:** Pediatria, Uso de medicamentos, Atenção primária à saúde

## Abstract

**Objetivo:**

Determinar a frequência de prescrições de medicamentos *off label* e não licenciados para pediatria na atenção primária à saúde em município de médio porte do Rio Grande do Sul, Brasil.

**Métodos:**

Estudo transversal, com coleta retrospectiva, que analisou prescrições a 326 pacientes emitidas de agosto a dezembro de 2012 em dois postos de saúde do município de Viamão. Foram incluídas todas as receitas de pacientes cujos prontuários ou fichas de atendimento estivessem disponíveis e completos em relação à data de atendimento, peso e data de nascimento. Foram classificadas como prescrições *off label* aquelas que, em relação à bula do medicamento, apresentavam dose diferente da recomendada, frequência de prescrição e/ou forma de administração diferente e idade inferior àquela indicada. Foi usada estatística descritiva, com frequências absolutas, médias e desvio padrão.

**Resultados:**

Durante o período estudado houve a prescrição de 731 medicamentos e houve frequência de 31,7% de medicamentos prescritos *off label*, especialmente anti-histamínicos e antiasmáticos (32,3% e 31,5%, respectivamente). O principal tipo de prescrição *off label* foi dose (38,8%), seguida de idade (31,5%) e de frequência de administração (29,3%). Com relação à prescrição *off label* de dose, foi mais frequente a sobredose (93,3%) do que a subdose (6,7%). Não foram encontradas prescrições de medicamentos não licenciados.

**Conclusões:**

O estudo mostrou que a prescrição *off label* é comum nas duas unidades estudadas. O percentual de prescrição *off label* observado foi superior ao relatado por estudos europeus feitos na atenção primária. Por outro lado, não foi observada prescrição de medicamentos não licenciados para crianças.

## Introdução

Considerando-se a carência de medicamentos para uso em crianças, especialmente para aquelas menores de dois anos, a prescrição de medicamentos *off label* se tornou uma prática rotineira tanto no âmbito hospitalar quanto ambulatorial e acarreta dúvidas aos prescritores e dispensadores com relação ao benefício para o paciente pediátrico.^1^^,^ 2

O termo *off label* se refere a medicamentos prescritos de forma diferente daquela orientada na bula ou compêndios oficiais, relativamente a dose, indicação, faixa etária, intervalo de administração ou forma de administração.^3^ A prescrição *off label* não é ilegal, não é necessariamente incorreta e está contemplada em vários protocolos pediátricos. A qualidade da terapia medicamentosa não está necessariamente relacionada ao *status* de licenciamento do medicamento. Entretanto, existem vários fatores clínicos, éticos e de segurança que deveriam ser considerados e não existem orientações para auxiliar a prescrição *off label*. A decisão sobre esse tipo de prescrição deve ser avaliada de acordo com a indicação clínica, as opções terapêuticas e a análise de risco-benefício. Além disso, é necessário que se tenha o consentimento do paciente ou responsável, com o cuidado de evitar a exposição de crianças a riscos desnecessários.^4^

Com relação ao conceito de medicamento não licenciado, alguns autores consideram que se refere àqueles que não apresentam registro no órgão de vigilância, ou que sejam preparações extemporâneas ou ainda aqueles que contêm ingredientes químicos não farmacológicos usados com objetivo terapêutico.^5^^-^ 8 Ferreira *et al*. ampliam o conceito de não licenciado para aqueles com registro, mas contraindicados para crianças.^9^

Existem estudos que caracterizam a extensão da prescrição *off label* na pediatria em hospitais no Brasil,^9^^-^ 11 mas pouco se sabe da prescrição ambulatorial no âmbito da atenção primária. Este estudo objetiva preencher essa lacuna, determinar a frequência de prescrições de medicamentos *off label* e não licenciados para pediatria, para subsidiar o desenvolvimento de ações de promoção do uso racional de medicamentos.

## Método

Trata-se de estudo transversal com coleta de dados retrospectiva, aprovado pelo Comitê de Ética em Pesquisa da Universidade Federal do Rio Grande do Sul (parecer n° 214.535) e autorizado pela Secretária Municipal de Saúde de Viamão. A coleta de dados foi feita em duas unidades básicas de saúde, Estratégia de Saúde da Família (ESF) Itapuã e Unidade de Referência (UR) Lomba do Sabão, em Viamão. Viamão é uma cidade da região metropolitana localizada a 20,6km da capital Porto Alegre, com uma área de 1.494,26km^2^ e 239.384 habitantes, de acordo com o Censo Demográfico do Instituto Brasileiro de Geografia e Estatística (IBGE) de 2010.^12^

Para estimar o tamanho da amostra, consideraram-se 3.759 atendimentos pediátricos nas unidades de saúde envolvidas em cinco meses (agosto a dezembro de 2012). Usou-se uma frequência esperada de prescrição de medicamentos *off label* de 20%.^4^^-^ 8 ^,^ 13 ^-^ 16 Para um intervalo de confiança de 95%, foi considerada uma variação de 20%+5%. Dessa forma, estimou-se que deveriam ser avaliadas no mínimo 231 prescrições.

Foram avaliadas cópias das prescrições retidas no dispensário da unidade após a dispensação, com medicamentos de pacientes até 12 anos atendidos pelos pediatras das respectivas unidades de saúde, de agosto a dezembro de 2012. Foram excluídas as prescrições de pacientes não vinculados à unidade. Prescrições de usuários da unidade cujos prontuários não foram encontrados ou estavam incompletos em relação às variáveis de interesse também não foram consideradas.

Os dados da prescrição eram coletados em uma tabela específica e completados com as informações do prontuário ou ficha de atendimento. Foram coletadas variáveis de interesse relacionadas com o paciente (idade, sexo e peso) e com as prescrições (total de itens, medicamentos prescritos, apresentações, forma farmacêutica, via de administração, frequência de administração e dose).

Para análise dos dados os pacientes foram divididos em quatro grupos de acordo com a idade: lactentes (0-2 anos), pré-escolares (>2-7 anos), escolares (>7-10 anos) e adolescentes (>10-12 anos). Os medicamentos prescritos foram classificados de acordo com o Bulário Eletrônico da Agência Nacional de Vigilância Sanitária (Anvisa)^17^ e, na falta de informações nesse site, na bula fornecida pelo fabricante em três classificações: de acordo com a especificação (idade, dose, frequência de administração e forma de administração de acordo com o especificado na bula); *off label (*medicamentos prescritos para idades diferentes das recomendadas, doses prescritas maiores ou menores do que as indicadas, frequências de administração diferentes das recomendadas e formas de administração discordantes das indicadas);^18^ e não licenciados para crianças (medicamentos para os quais não havia informação ou que eram contraindicados para crianças,^9^ registro e bula só contemplavam o uso adulto).

Foi usada a Anatomical Therapeutic Chemical Classification (ATC)^19^ para possibilitar a análise dos medicamentos por classes terapêuticas. Os dados foram organizados em planilha do Microsoft Office Excel 2007 e analisados no programa informatizado SPSS 18.0. Foi usada estatística descritiva com frequências absolutas, médias e desvio padrão.

## Resultados

Receitas de 705 pacientes pediátricos foram retidas no período de estudo nas unidades selecionadas, em geral mais de uma receita por paciente. Somente 326 dessas receitas foram incluídas no estudo, pois 379 prontuários não foram localizados ou estavam incompletos. Considerando-se a unidade de atendimento, 203 (62,3%) pacientes estudados pertenciam à Estratégia de Saúde da Família (ESF) Itapuã e os outros 123 (37,7%) à Unidade de Referência (UR) Lomba do Sabão. Das 326 crianças, 56,4% eram do sexo masculino. Houve maior frequência de lactentes 142 (43,3%), seguidos dos pré-escolares 103 (31,6%). Escolares 55 (16,9%) e adolescentes 27 (8,3%) foram minoria, somaram 25,2% dos pacientes. Dentre os lactentes (0-2 anos), 89 (63%) tinham até 12 meses e 53 (37%) de 13 meses a dois anos. O número de medicamentos variou de um a oito, com uma média de 2,2±1,4 medicamentos por paciente. De acordo com a análise das prescrições, 95,4% de todos os medicamentos prescritos pertenciam à lista municipal de medicamentos essenciais (Remume).

Foram prescritos 39 princípios ativos diferentes (30 isolados e nove combinações) em diferentes apresentações. Os medicamentos prescritos em maior frequência foram: Paracetamol 88 (11,8%); soro fisiológico nasal 81 (11,1%); Loratadina 80 (10,3%); Amoxicilina 66 (8,3%); Prednisolona 60 (8,2%) e Salbutamol *spray* oral 53 (7,3%). A tabela 1 descreve a frequência de prescrição por classe terapêutica.

**Tabela 1:** Classes terapêuticas dos medicamentos prescritos e frequência de prescrição

Grupo ATC	Classe	Medicamentos (número de prescrições)	Prescrições n (%)
N02	Analgésicos, antitérmicos	Paracetamol (88), dipirona (7), ibuprofeno (26) a	121(16,6%)
R03	Fármacos usados nas doenças respiratórias obstrutivas	Beclometasona (40), cromoglicato dissódico (1), fenoterol (18), salbutamol (53), budesonida (1)	113 (15,5%)
R01	Preparações nasais	Budesonida (4), cloreto de sódio (81), beclometasona (24)	10 (14,9%)
R06	Anti‐histamínicos de uso sistêmico	Loratadina (80), dexclorfeniramina (8), bronfeniramina (1)	89 (12,2%)
J01	Antimicrobianos de uso sistêmico	Amoxicilina (66), cefalexina (6), sulfametoxazol + trimetoprima (2), metronidazol (1)	75 (10,3%)
H02	Corticosteroide de uso sistêmico	Prednisolona (60), prednisona (12)	72 (9,9%)
P02	Antiparasitários, anti‐helmínticos	Albendazol (19), ivermectina (1), mebendazol (12)	32 (4,4%)
A03	Fármacos usados nas desordens gastrintestinais	dimeticona (18), metoclopramida (7), bromoprida (4)	29 (4,0%)
B03	Antianêmicos	Sulfato ferroso (21)	21 (2,9%)
D07	Corticosteroide de uso tópico	Dexametasona (17), desonida (1)	18 (2,5%)
D01	Antifúngico de uso tópico	miconazol (11)	11 (1,5%)
A011	Vitaminas	Vitamina A + D (10)	10 (1,4%)
D06	Antimicrobianos para uso tópico	Neomicina + bacitracina (8)	8 (1,1%)
S02	Fármacos de uso otológico	Borato de 8‐hidroxiquinolina trolamina (5), neomocina + polimixina + hidrocortisona (1)	6 (0,8%)
A01	Preparações estomatológicas	Nistatina (5)	5 (0,7%)
P03	Ectoparasiticidas/escabicidas	Permetrina (4)	4 (0,6%)
A07	Antidiarreicos, agentes antimicrobianos	Sais de reidratação oral (3), *Saccharomyces boulardii* (1)	4 (0,6%)
A06	Fármacos usados na constipação	Lactulose (2)	2 (0,3%)
M01	Anti‐inflamatórios	Nimesulida (1)	1 (0,1%)
D02	Emolientes e protetores	Óxido de zinco (pasta d’água) (1)	1 (0,1%)

a Ibuprofeno i prescrito como analgésico e antitérmico

ATC, Anatomical Therapeutic Chemical Classification.

Encontramos 731 medicamentos prescritos. Não houve prescrição de medicamentos não licenciados para crianças. Todos os medicamentos prescritos eram de uso adulto e pediátrico. Do total, 232 (31,7%) prescrições eram *off label* e 13 (0,02%) não tinham especificação da dose prescrita, somente o nome do princípio ativo. Os medicamentos sem especificação de dose foram: vitamina A+D e Permetrina (em nove [1,2%] e em quatro [0,6%] crianças, respectivamente). A classificação dos medicamentos prescritos está resumida na tabela 2.

**Tabela 2:** Classificação dos medicamentos prescritos em relação a sua especificação fornecida pela bula

Classificação	Frequência n (%)
De acordo com a especificação	486 (66,5%)
Não licenciados	0
Prescrição *off label*	232 (31,7%)
Não foi possível classificar a	13 (1,8%)
Total de medicamentos prescritos	731 (100%)

a Não foi possível classificar, pois na prescrição não constava apresentação farmacêutica.

Dentre as prescrições *off label*, observaram-se os seguintes tipos e frequências: *off label* de dose, 90 (38,8%), seguida de idade, 73 (31,5%) e de frequência de administração 68 (29,3%). Com relação ao *off label* relacionado à dose, foi mais prevalente a sobredose do que a subdose: 84 (93,3%) *vs*. 6 (6,7%) prescrições, respectivamente.

Os medicamentos prescritos *off label* são apresentados na tabela 3, por classes terapêuticas. Dentre esses se destacam: Loratadina, Salbutamol spray oral, Fenoterol e Dimeticona.

**Tabela 3:** Tipo de uso *off label* por classe terapêutica

Grupo ATC	Classe	Dose (n=90)	Idade (n=73)	Intervalo de administração (n=68)	Forma de administração (n=1)
R06	Anti‐histamínicos de uso sistêmico	17 (7,3%)	22 (9,5%)	36 (15,5%)	
R03	Fármacos usados nas doenças respiratórias obstrutivas	28 (12,1%)	30 (12,9%)	15 (6,5%)	
J01	Antimicrobianos de uso sistêmico	25 (10,8%)			
A03	Fármacos usados nas desordens gastrintestinais	2 (0,9%)	7 (3,0%)	15 (6,5%)	
R01	Preparações nasais	1 (0,4%)	8 (3,5%)	1 (0,4%)	
N02	Analgésicos, antitérmicos	5 (2,2%)	3 (1,3%)		
B03	Antianêmicos	4 (1,7%)			1 (0,4%)
H02	Corticosteroide de uso sistêmico	3 (1,3%)			
P02	Antiparasitários, anti‐helmínticos	2 (0,9%)			
M01	Anti‐inflamatórios	1 (0,4%)	2 (0,9%)	1 (0,4%)	
S02	Fármacos de uso otológico	2 (0,9%)	1 (0,4%)		

Porcentagem sobre o total de prescrições *off label* (232). ATC, Anatomical Therapeutic Chemical Classification.

A Loratadina foi o terceiro medicamento mais prescrito e teve uma frequência de prescrição *off label* de 85,3%, 53,1% por frequência de administração, 25% para idade inferior à recomendada e 21,9% por sobredose. Considerando as prescrições *off label* por frequência de administração, 19 (55,9%) foram *off label* também em dose, ou seja, dois tipos de uso discordante do recomendado.

O Salbutamol, cuja frequência de prescrição foi 7,3% (53), foi prescrito *off label* em 100% das prescrições: indicado para faixa etária inferior à recomendada em 27 (50,9%) e para uso em doses acima do recomendado pela bula em 26 (49,1%) casos.

O Fenoterol teve 18 prescrições. Somente uma delas estava de acordo com a bula, as outras 17 (94,4%) foram classificadas como *off label*. Dessas 17, 15 (88,2%) eram *off label* por frequência de administração acima da recomendada e duas (11,8%) devido à dose acima da recomendada. As duas prescrições com dose acima também tinham frequência de administração acima da recomendada.

A Dimeticona, medicamento fora da lista municipal de medicamentos essenciais, foi prescrita em 17 ocasiões, 16 delas (94,1%) *off label*: 14 por frequência de administração acima da recomendada e duas vezes por sobredose. Nos dois casos de sobredose, também houve prescrição de frequência de administração incorreta.

Na análise dos medicamentos prescritos *off label* por faixa etária, chama atenção a prescrição de Loratadina e Dexclorfeniramina para lactentes, idade na qual tais medicamentos não são recomendados. No grupo dos pré-escolares, destacam-se as prescrições de Salbutamol e Amoxicilina em doses acima das recomendadas. Nas faixas etárias correspondentes aos escolares e adolescentes, a sobredose do Salbutamol foi o que mais se destacou.

## Discussão

O estudo não identificou a prescrição de medicamentos não licenciados. Outros autores encontraram percentuais que variaram de 0,3 a 16,8%.^5^^,^ 14 ^,^ 18 A ausência de medicamentos não licenciados é relevante e pode estar relacionada à elevada (95,4%) adesão dos pediatras do município de Viamão à lista de medicamentos essenciais do município (Remume). Um percentual inferior de adesão à Remume (76,4%) foi observado em estudo feito em oito municípios de três estados brasileiros.^20^ O uso de listas de medicamentos essenciais é uma medida recomendada pela Organização Mundial da Saúde para promover o uso racional de medicamentos.^21^ A disponibilidade de um arsenal terapêutico menor e que leva em consideração as necessidades de saúde da maioria da população pode reduzir as chances de uso de produtos não licenciados.

Por outro lado, a prescrição de medicamentos *off label* na atenção primária de Viamão mostrou-se elevada, com uma frequência de 31,7%, acima da faixa encontrada em outros estudos de base populacional europeus: Escócia (24,6%), Inglaterra (16%), Holanda (20,3%), Estônia (31%), Itália (17%) e França (29%).^6^^,^ 8 ^,^ 13 ^,^ 15 ^,^ 22 ^,^ 23 Os principais tipos de prescrição *off label* nesse estudo foram dose (38,8%), idade (31,5%) e frequência de administração (29,3%). Os estudos europeus supracitados indicaram, de maneira similar, dose e idade como principais tipos de uso *off label*, nessa ordem. A frequência de administração dos medicamentos não figurou entre os tipos de *off label*, o que, provavelmente, se deve ao fato de que outros autores avaliaram a dose e a frequência de dose em conjunto e classificaram os casos fora da especificação como *off label* de dose.

A sobredosagem foi mais frequente, diferentemente do relatado por outros autores que observaram maior número de subdoses, especialmente para a classe dos antimicrobianos de uso sistêmico.^13^^,^ 14 ^,^ 24 Houve prevalência de 10,3% de prescrições de antimicrobianos, que representaram 10,8% do total de prescrições *off label*, majoritariamente por sobredose*.* Esse resultado mostra uma tendência diversa de outros estudos que associam a subdose à dificuldade dos prescritores de adaptar a dose à idade da criança, ou seja, de saber a idade e situações certas em que as doses devem ser incrementadas.^24^ A amoxicilina, o antimicrobiano mais prescrito neste estudo, tem uma orientação de dose na bula inferior a outras fontes, como o Formulário Terapêutico Nacional.^25^ Os dados sugerem desatualização da bula do medicamento publicada no bulário eletrônico da Anvisa e reforçam a necessidade de atualização constante dessa fonte de informação por parte dos fabricantes e a revisão da licença do produto junto à agência. Outra medida relevante seria o estabelecimento de Comissão de Farmácia e Terapêutica municipal, com vistas à elaboração de protocolos de uso dos medicamentos da lista de acordo com estudos atualizados, para respaldar o prescritor e propiciar o uso racional dos medicamentos. Vale lembrar que a qualidade da terapia medicamentosa não está necessariamente relacionada ao status de licenciamento do medicamento.

Os medicamentos mais prescritos *off label* foram os antiasmáticos e os anti-histamínicos de uso sistêmico (tabela 3). Essas classes de medicamentos estão entre as mais comumente prescritas para pediatria na atenção primária.^6^^,^ 16 Neste estudo, a Loratadina foi prescrita *off label* em 85,3% dos casos por frequência de administração (53,1%), por idade inferior à recomendada (25%) e por sobredose (21,9%). Considerando-se a meia-vida plasmática do medicamento de 17-24 horas,^25^ não haveria necessidade ou indicação para múltiplas doses diárias, como observado neste estudo. O uso de anti-histamínicos de forma não apropriada para a idade foi relatado em revisão bibliográfica recente, que mostrou variação de 6,5% a 43%.^26^ Da Costa *et al*. apontam a Loratadina como um dos medicamentos problema em pediatria devido à restrição para idade abaixo de dois anos.^1^ A dose usual para a Loratadina é 5 mg para crianças de 2-6 anos e abaixo de 30 kg e de 10 mg uma vez ao dia acima de seis anos e em adultos.^25^^,^ 26 O estudo mostrou sobredose nas prescrições de Loratadina. Somente um estudo foi encontrado que sustentava dose maior do que a apresentada na bula para tratamento de asma alérgica.^27^ Considerando-se que essa indicação não é confirmada nas Diretrizes da Sociedade Brasileira de Pneumologia e Tisiologia para o Manejo da Asma de 2012, fica evidente a necessidade de educação continuada dos profissionais e padronização de doses com base em estudos de ensaio clínico e, na falta desses, de estudos observacionais.^28^

Houve uma prevalência de prescrições *off label* para o tratamento das doenças respiratórias de 31,5% (tabela 3), destacaram-se, dentre os medicamentos, o Salbutamol *spray* e o Fenoterol solução para nebulização. O tratamento da asma em crianças é um desafio e, muitas vezes, uma sobreposição entre sibilância recorrente e fenótipos de asma ocorre, tornando o diagnóstico e as decisões terapêuticas controversas.^28^ As Recomendações da Sociedade Brasileira de Pneumologia e Tisiologia (SBPT) diferem das recomendações da bula do medicamento Salbutamol *spray*. As diretrizes indicam o uso em crianças acima de cinco anos e com doses maiores do que aquelas indicadas pelo fabricante, mesmo com as possíveis limitações para uso correto do dispositivo nessa faixa etária. A bula do medicamento salienta a dificuldade do uso correto do dispositivo em crianças abaixo de sete anos, sem ressalvas para o uso acima dessa faixa etária. Considerando-se o grande número de internações e os riscos do não tratamento de uma crise asmática (a asfixia é a principal causa de óbito na quase totalidade dos casos),^28^ a adaptação da dose adulta para crianças é uma prática recorrente baseada no conhecimento do clínico, mas com pouca documentação pelos médicos brasileiros.^25^ Embora o benefício clínico desses medicamentos no tratamento da asma aguda seja bem documentado, há grande variabilidade nas doses usadas, a maioria dessas é baseada em opinião de especialistas, consensos clínicos ou estudos com um número limitado de pacientes. Poucas evidências apoiam com precisão as doses a serem usadas.^28^

O uso de Salbutamol, um agonista beta adrenérgico, em doses elevadas está associado a tremores, agitação, hipocalemia e arritmias cardíacas. Existe evidência de que o tratamento com Salbutamol desloca a regulação autonômica cardiovascular para um novo nível, caracterizado por uma maior capacidade de resposta simpática e tolerância leve do receptor β_2_. Com base nessa evidência, o abuso de Salbutamol pode ser substrato para a fibrilação atrial.^29^

O determinante mais importante da dosagem diária é o julgamento clínico da resposta do paciente ao tratamento. O médico deve monitorar a resposta do paciente e ajustar a dose de acordo com o nível de controle da asma. As definições de doses baixas, médias ou altas são baseadas em estudos farmacocinéticos e farmacodinâmicos dos fabricantes, que raramente são baseados em curvas dose-resposta. As mesmas variam de acordo com o dispositivo usado e devem ser avaliadas individualmente.^28^

Chama a atenção o fato de que algumas prescrições não apresentavam posologia, apenas o nome do medicamento. Para o paciente a prescrição representa um importante apoio no tratamento e para ser efetiva deveria apresentar os itens básicos, dentre eles a posologia.^30^

A maior parte dos medicamentos usados por crianças é prescrita na atenção primária e os pediatras ou clínicos gerais fazem uso de um número relativamente pequeno de medicamentos para solucionar os problemas mais frequentes. O elenco reduzido de medicamentos facilita a elaboração de protocolo clínico municipal, construído de forma multidisciplinar na Comissão de Farmácia e Terapêutica. O que contrasta com a atenção secundária, na qual o número de crianças é menor em relação à população geral, mas um número muito maior de medicamentos é prescrito para tratar condições raras e de maior gravidade.^13^

A extrapolação dos resultados deste estudo deve ser feita com cautela, uma vez que os dados se referem a duas unidades de saúde, contemplam um período de cinco meses e metade das prescrições não pôde ser avaliada por falta de informações. Os hábitos de prescrição podem variar de acordo com a formação dos pediatras, a existência de protocolos ou mesmo de rotinas de serviço. São necessários estudos que envolvam um maior número de unidades de saúde para se estabelecer com mais precisão a extensão da prescrição *off label* em nível municipal ou regional. Além disso, devem ser consideradas as limitações inerentes aos estudos retrospectivos. As informações foram coletadas de receitas retidas nas farmácias e dos prontuários manuais. O registro incompleto do nome de pacientes e diferentes critérios de catalogação dos prontuários determinou a exclusão de alguns casos. A coleta de dados retrospectiva também impossibilitou a análise de uso *off label* por indicação, uma vez que esse dado raramente foi encontrado no prontuário ou nas fichas de atendimento. Deve-se considerar ainda que nas farmácias dessas unidades ficam retidas somente as receitas aviadas. Os pacientes podem ter recebido outras receitas com medicamentos sujeitos a controle especial, não pertencentes à Remume, ou do componente especial ou especializado da assistência farmacêutica. Apesar dessas limitações, este estudo traz contribuições, uma vez que os dados brasileiros referentes a uso de medicamentos não licenciados e *off label* na pediatria, até o momento, se restringem ao âmbito hospitalar.

As dificuldades relacionadas à pesquisa com crianças propiciam a prescrição de medicamentos *off label*. Tal prática, apesar de não ser ilegal, gera insegurança em relação aos possíveis efeitos adversos em uma população com características peculiares como a pediátrica. O presente estudo mostrou que essa prática é comum na atenção primária à saúde em uma cidade do Rio Grande do Sul, a exemplo de pesquisas em cidades europeias. Espera-se que os resultados obtidos possam contribuir para o planejamento de ações no sentido de apoiar os prescritores e proporcionar maior segurança no uso de medicamentos por pacientes pediátricos.
